# A Conceptual Thematic Framework of Psychological Adjustment in Caregivers of Children with Craniofacial Microsomia

**DOI:** 10.1177/10556656241245284

**Published:** 2024-04-08

**Authors:** Nicola M. Stock, Bruna Costa, Jade Parnell, Alexis L. Johns, Canice E. Crerand, Kristin Billaud Feragen, Laura P. Stueckle, Angela Mills, Leanne Magee, Matthew Hotton, Melissa Tumblin, Amy Schefer, Amelia F. Drake, Carrie L. Heike

**Affiliations:** 1 Centre for Appearance Research, University of the West of England, Bristol, UK; 2 Division of Plastic and Maxillofacial Surgery, Children's Hospital Los Angeles, Los Angeles, CA, USA; 3 Nationwide Children's Hospital, Columbus, OH, USA; 4 Centre for Rare Disorders, Oslo University Hospital, Oslo, Norway; 5 Center for Clinical and Translational Research, Seattle Children's Research Institute, Seattle, WA, USA; 6 Buerger Center for Advanced Pediatric Care, Children's Hospital of Philadelphia, Philadelphia, PA, USA; 7 Oxford Institute of Clinical Psychology Training and Research, University of Oxford, Oxford, England; 8 Department of Otolaryngology/Head and Neck Surgery, University of North Carolina at Chapel School of Medicine, Chapel Hill, NC, USA

**Keywords:** craniofacial microsomia, evidence-based practice, familial adjustment, feeding, maternal factors, mental health support, parental perception, psychosocial adjustment, quality of life, team care

## Abstract

**Objective:**

Children with craniofacial microsomia (CFM) have complex healthcare needs, resulting in evaluations and interventions from infancy onward. Yet, little is understood about families’ treatment experiences or the impact of CFM on caregivers’ well-being. To address this gap, the NIH-funded ‘Craniofacial microsomia: Accelerating Research and Education (CARE)’ program sought to develop a conceptual thematic framework of caregiver adjustment to CFM.

**Design:**

Caregivers reported on their child's medical and surgical history. Narrative interviews were conducted with US caregivers (*n *= 62) of children aged 3-17 years with CFM. Transcripts were inductively coded and final themes and subthemes were identified.

**Results:**

Components of the framework included: 1) Diagnostic Experiences, including pregnancy and birth, initial emotional responses, communication about the diagnosis by healthcare providers, and information-seeking behaviors; 2) Child Health and Healthcare Experiences, including feeding, the child's physical health, burden of care, medical decision-making, surgical experiences, and the perceived quality of care; 3) Child Development, including cognition and behavior, educational provision, social experiences, and emotional well-being; and 4) Family Functioning, including parental well-being, relationships, coping strategies, and personal growth. Participants also identified a series of “high” and “low” points throughout their journey and shared their priorities for future research.

**Conclusions:**

Narrative interviews provided rich insight into caregivers’ experiences of having a child with CFM and enabled the development of a conceptual thematic framework to guide clinical care and future research. Information gathered from this study demonstrates the need to incorporate evidence-based psychological support for families into the CFM pathway from birth onward.

## Introduction

Craniofacial microsomia (CFM) is a congenital condition characterized by underdevelopment of the facial structures, and most commonly affects the ear and mandible. CFM is associated with wide clinical variability and the cause is unknown for most children.^1^ CFM-related features can affect feeding, compromise the airway, limit facial movement, impair hearing, and alter facial appearance.^1-3^ Although the condition is relatively rare, it is the third most common diagnosis requiring interdisciplinary craniofacial team care.^1-3^ Multidisciplinary team care is recommended to address these complex health needs and coordinate evaluations and interventions to ensure the best holistic outcomes for the child and the family.^2,3^

The birth of a child with medical and/or developmental needs can pose a range of psychological and social challenges for caregivers and families.^4,5^ These stressors may be related to caregivers’ experience at the time of diagnosis, developmental transitions, the ongoing healthcare needs of their child, and changes in the child's health or need for hospitalizations.^6^ As a result of these challenges, caregivers of children with medical needs have reported significantly greater levels of stress, anxiety and depression, as well as poorer physical health than parents of unaffected children.^4,5,7,8^ Similar patterns have been observed in families of children with congenital craniofacial conditions, with caregivers reporting less favorable anxiety, depression and stress scores compared to general population norms.^9-11^ Parental adjustment to the demands of their child's health condition is crucial for their own long-term psychological health, as well as the emotional, social, and cognitive development of the child, and family functioning as a whole.^4^

Despite the long-term and multifaceted nature of treatment and the anticipated impact of CFM on the quality of life of affected families, relatively little is known about caregivers’ experiences. A comprehensive review of the psychosocial literature pertaining to CFM^12^ identified a handful of studies examining aspects of the caregiver journey. In these studies, caregivers frequently reported a lack of information at the time of birth, described a wide range of positive and negative responses to their child's diagnosis, and wanted to know more about the etiology of CFM.^13-16^ Across home, community, and medical settings, caregivers identified the burden of care, treatment decisions, social stigma, and accessing and navigating school systems to have a notable impact on their own well-being.^13,15-17^ Caregivers used a range of coping strategies to overcome these challenges, including seeking support from family, completing healthcare provider consultations, practing their faith, and comparing themselves to others whom they perceived to be less fortunate.^14^

While these studies provide important insights, an in-depth understanding of the caregiver CFM journey is lacking. Additionally, CFM treatment protocols vary widely across craniofacial centers, and no standardized psychological support is currently available. Particularly in the case of rare conditions, qualitative research can provide unique knowledge of what it is like to have a child with additional needs, families’ experiences of healthcare, and the adequacy of current service provision in addressing caregivers’ concerns. Yet, qualitative research is significantly absent from the CFM literature, particularly when compared to other health conditions.^18^ In response to these gaps, the ‘Craniofacial microsomia: Accelerating Research and Education (CARE)’ research program was developed to better understand the psychological health and healthcare experiences of individuals with CFM and their caregivers, and to identify opportunities for psychological intervention and improved healthcare provision.^19^ The aim of the current study was to develop a conceptual thematic framework of psychological adjustment to CFM in caregivers to inform future research and practice.

## Methods

### Design

This study is part of the larger CARE research grant.^19^ In the initial phase of this grant, remote, individual, narrative interviews with caregivers of children with CFM were conducted.

### Procedure

#### Ethical Considerations

Ethical approval was granted by the Institutional Review Board at Seattle Children's Hospital, USA. All documents were subsequently ratified by the Faculty Research Ethics Committee at the University of the West of England (UWE), UK. Before any study procedures were performed, informed consent was collected from participants over the phone, via videoconference, or in-person, and in a location that allowed for privacy. IRB approval included a waiver of documentation of consent, and therefore all participants consented verbally. The date of consent was documented in the tracking database.

#### Recruitment

CFM is a broad term that includes the following clinical diagnoses: microtia, hemifacial microsomia, Goldenhar syndrome, and Oculo-Auriculo-Vertebral Spectrum.^19^ English and Spanish-speaking caregivers with children who met the research criteria for CFM established by the Facial Asymmetry Collaborative for Interdisciplinary Assessment and Learning (FACIAL) network^1^ were eligible to participate in the study. Minimal inclusion criteria included a diagnosis of microtia and/or one of the following combinations of features: facial asymmetry and preauricular tag(s); facial asymmetry and facial tag(s); facial asymmetry and epibulbar dermoid; facial asymmetry and a lateral oral cleft (ie, macrostomia); preauricular tag(s) and epibulbar dermoid(s); preauricular tag and a lateral oral cleft; facial tag(s) and epibulbar dermoid; a lateral oral cleft and epibulbar dermoid(s). Exclusion criteria included a diagnosis of a specific syndrome, such as Treacher Collins, Townes-Brocks or Nager, and/or a major medical condition not associated with CFM that participants felt had a more substantial impact on their health, such as cancer. Participants were recruited across the United States (US). Online recruitment took place via advertisements on social media, blogs, newsletters, and emails posted by collaborating CFM-related support and advocacy groups. In-person recruitment was performed at hospital-affiliated craniofacial and specialty clinics, community events hosted by CFM-related advocacy groups, word-of-mouth, and direct contact with participants of prior CFM research studies who had expressed an interest in future research. Enrollment was actively monitored to ensure the inclusion of caregivers of children with CFM that represented the large US geographic regions, the diverse range of healthcare needs associated with CFM, and the full spectrum of ages (3-17 years) selected for this study.

#### Personnel Training

The research teams at each site received comprehensive training during the first six months of the study. Training included: a) regulatory requirements; b) participant approach, recruitment, and consent; c) medical and surgical history, and d) photograph acquisition. All narrative interviewers (*n *= 5) were trained in qualitative interviewing techniques and most completed a minimum of two practice interviews, which were checked for quality and fidelity prior to study commencement, in addition to receiving feedback from caregiver advocates.

#### Medical and Surgical History

Participating caregivers were asked questions by telephone regarding their race, ethnicity, health insurance status, education, occupation, pregnancy (biologic mothers only), and family health history. Participants were also asked about their child's demographic characteristics and clinical history, including medical care, diagnostic and screening tests, interventions, healthcare subspecialists, and surgeries. The phone calls for gathering medical and surgical history lasted an average of 40 min. Separate appointments were scheduled for the narrative interviews.

#### Photographs

Caregivers provided standardized 2D facial photographs of their children. Participants used a simplified version of the photographic protocol from the FACIAL network and used their smartphone or a camera to obtain frontal views (smiling and neutral), lateral views (right and left), and close up views of each ear. After completing the narrative interview (see below), participants received an email with a personalized link to a secure database that had example images for each view. Photographs were incorporated into the phenotyping protocol described below.

#### Narrative Interview

Narrative interviews were conducted in English or Spanish via telephone (*n *= 7) or a teleconference platform (*n *= 55), according to the participants’ choice. The narrative interview method used an adapted version of the ‘Life Story’ interview, which involves asking participants to divide their CFM story into “chapters” based on experiences that were meaningful to them. In contrast to semi-structured interviews, which predetermine areas of content to be discussed, the narrative approach allows participants to freely identify and discuss the subject areas they deem to be salient in their lived experiences.^20,21^ Interviewers asked questions at the completion of each chapter to clarify and/or elaborate on aspects of participants’ narratives. Participants were also asked to identify a “low” point (a particularly difficult aspect) and a “high” point (a particularly positive aspect) for each chapter of their story. Finally, interviewers asked how participants’ experiences could have been improved, their thoughts about priorities for future research, and their reflections on the narrative interview process.

### Analysis

#### Medical, Surgical and Phenotypic Data Integration

All phenotypic coding was performed by a pediatric craniofacial specialist. Images were classified using the PAT-CFM tool,^22^ which includes a check-box format adapted from the Orbit Mandible Ear facial Nerve and Soft tissue (OMENS) system. To categorize the participant's phenotype, data were used from the PAT-CFM ratings, the medical history information provided by caregivers, and medical chart abstractions. Data from all sources were integrated and reviewed to establish the phenotype by feature for each participant and entered into an Access database.

#### Qualitative Interview Data

A total of 62 narrative interviews with caregivers were completed, including 50 completed in English (80.6%) and 12 in Spanish (19.4%). Interviews ranged from 30 to 191 min in length (M = 83 min). Interviews conducted in English were transcribed by an external individual transcriptionist. Interviews conducted in Spanish were transcribed and translated by a professional transcription company. Analysis of caregiver interviews carried out in English was divided into three categories to align with broad developmental phases (birth to 3 years, 4-11 years, and 12-17 years). Given the volume of data collected, transcripts were analyzed by a total of 6 researchers. This included 5 researchers for the English interviews, whose time was divided across the three categories, and 2 researchers for the Spanish interviews. The UWE PI had oversight throughout the process. All researchers were trained in qualitative analysis, and 3 were senior investigators with extensive qualitative experience. The analytic process was informed by Braun and Clarke's coding reliability approach to Thematic Analysis.^23^ All researchers became familiar with caregiver narratives through multiple readings of all transcripts, and individually and inductively coded a percentage of the transcripts. Researchers then met to discuss their initial findings, identify any discrepancies, and refine the coding method. During this iterative process, which included regular meetings between researchers to review code content, “codebooks” were produced, and codes were further developed and refined. Interviews conducted in Spanish were analyzed separately, building on the existing English codebooks. Reflections on the analytical process were discussed during team meetings and key decisions were documented. Preliminary themes were reviewed by the UWE PI and discussed until consensus was reached. Final themes and subthemes were grouped into a conceptual thematic framework by the UWE PI, and the framework was approved by all authors.

## Results

### Participant Characteristics

As described in Table 1, participants included 57 mothers (91.9%) and 5 fathers (8.1%), of whom 6 were adoptive parents (9.7%). Caregivers had a mean age of 40.2 years (SD = 11.9), were primarily White (61.3%), were married/cohabiting (77.4%), and had completed college (54.8%). Participants were recruited from 23 US states and most families lived in the western region (61%). Families had private health insurance (56.5%) or public health insurance (43.5%). The mean age of participants’ children at the time of interview was 10.4 years (SD = 4.4), and just over half of the children were male (51.6%). An illustration of the characteristics of the study population is provided in the Supplementary Material.

**Table 1.** Characteristics of Participating Caregivers of Children with Craniofacial Microsomia.

	**N (%)** (N = 62)	**US Census Bureau (%)**
Age at interview in years (mean, SD)	40.2 (11.9)	
Gender		
Female	57 (91.9)	
Male	5 (8.1)	
Adoptive Parents		
No	56 (90.3)	
Yes	6 (9.7)	
Interview Language		
English	50 (80.6)	
Spanish	12 (19.4)	
Interview Platform		
Video Conference	55 (88.7)	
Telephone	7(11.3)	
Race/Ethnicity		
White (not Hispanic/Latinx)	38 (61.3)	58.9
Hispanic/Latinx	16 (25.8)	19.1
Multi-Racial	6 (9.7)	3.0
Asian	1 (1.6)	6.3
Native Hawaiian/Pacific Islander	1 (1.6)	0.3
Black or African American	0 (0)	13.6
American Indian/Alaska Native	0 (0)	1.3
Insurance Status		
Private	35 (56.5)	65.6
Public	27 (43.5)	36.1
Uninsured	0 (0)	7.9
Marital Status		
Married	46 (74.2)	47.6
Divorced	5 (8.1)	9.5
Never Married (single)	5 (8.1)	34.0
Living with Partner	2 (3.2)	
Widowed	2 (3.2)	5.7
Separated	1 (1.6)	1.7
Unknown	1 (1.6)	
Education Status		
Unknown	1 (1.6)	
<12 years (no high school diploma)	8 (12.9)	
12 years high school/diploma/GED	4 (6.5)	High school graduate or higher 89.1
Some college/associate degree	15 (24.2)	
Completed university/college	34 (54.8)	
Employment Status	52 (82.3)	
Working now	7 (11.3)	63.0
Stay-at-home parent	4 (6.5)	
Other		
Region		
West	38 (61.3)	
Midwest	11 (17.7)	
Northeast	5 (8.1)	
Southeast	5 (8.1)	
Southwest	3 (4.8)	

### Medical and Surgical History of Caregiver's Children with CFM

Although no caregivers were given a definitive diagnosis for their child at the time of birth, most children had received a formal craniofacial diagnosis by 6 months of age (83.9%). The most common diagnostic term (or combination of terms) reported by caregivers (Table 2) included: microtia (88.7%), hemifacial microsomia (45.2%), CFM (43.5%), and Goldenhar syndrome (21.0%). Phenotypic subgroups included: microtia only (12.9%), microtia with mandibular hypoplasia (71%), and other combinations of CFM-related features (16.1%). Specific CFM features reported by caregivers included microtia (98.4%), external ear canal atresia (82.3%), and mandibular hypoplasia (72.6%). Other common CFM-related craniofacial features included: facial nerve palsy (24.2%), epibulbar dermoids (12.9%), and lateral oral cleft (11.3%). Extracranial malformations were present in 37.1% children and included congenital heart anomalies (21.0%) and musculoskeletal anomalies (17.7%). Nearly all children had been seen by a subspecialist (98.4%), with an average of 9.6 (SD = 4.8) subspecialty providers per child. Most children had been seen by a craniofacial team (74.2%) and had an average age of 1 year (SD = 1.39) at the time of their first team visit.

**Table 2.** Characteristics of the Child.

	**N** (N = 62)	**%**
Age at interview in years (mean, SD)	10.4 (4.4)	
Gender		
Male	32	51.6%
Female	30	48.4%
Phenotype Category		
Microtia + mandibular hypoplasia	44	71.0%
Microtia only	8	12.9%
Other combinations of CFM-related features	10	16.1%
Phenotype Features (may have more than one)		
Microtia	61	98.4%
Atresia	51	82.3%
Mandibular hypoplasia	45	72.6%
Extracranial anomalies	23	37.1%
Types of anomalies (may have more than one)		
Congenital heart defect	13	21.0%
Spinal anomaly	11	17.7%
Kidney anomaly	4	6.5%
Pulmonary anomaly	1	1.6%
Other anomaly	12	19.4%
Facial nerve palsy	15	24.2%
Epibulbar dermoid	8	12.9%
Lateral oral cleft	7	11.3%
Eyelid coloboma	4	6.5%
Cleft palate	4	6.5%
Cleft lip	1	1.6%
How many days after birth was patient discharged? (mean, SD)	11.9 (33.8)	
CFM diagnosis terms used by caregivers (can be more than one)		
Microtia	55	88.7%
Hemifacial microsomia (HFM)	28	45.2%
Craniofacial microsomia (CFM)	27	43.5%
Goldenhar Syndrome	13	21.0%
Oculo Auriculo Vertebral Syndrome (OAVS)	5	8.1%
Other CFM diagnosis	2	3.2%
Age at diagnosis in months (mean, SD)	2.9 (8.8)	
< 6 mos	52	83.9%
≥6 mos	9	14.5%
Unknown	1	1.6%
Seen at a craniofacial clinic		
No	13	21.0%
Yes	46	74.2%
Unknown	3	4.8%
Hearing aid use (ever)		
Yes	49	79.0%
Age at first use of hearing aid in years (mean, SD)	2.5 (3.5)	
Seen by a subspecialty provider	61	98.4%
Number of providers seen (mean, SD)	9.6 (4.8)	
Child currently in school	60	96.8%
Type of classroom		
General education without additional academic support	41	68.3%
General education w/additional academic support	7	11.7%
Special education/special day class all day	2	3.3%
Other classroom placement	4	6.7%
Unknown	8	13.3%
Any intervention services (current or past)	55	88.7%

As seen in Table 3, the majority of children had undergone surgery (96.8%) with a median number of surgeries per child of 4 (range 1-21). The most common surgeries were ear reconstruction (38.7%), removal of preauricular and/or facial tags (38.7%), and placement of tympanostomy tubes (32.3%). Airway surgeries included adenoidectomy and/or tonsillectomy (27.4%) and tracheostomy (4.8%). Many children had also undergone dental extractions and/or restorations (25.8%).

**Table 3.** Surgeries Undergone by Participants’ Children.

	**N = 62**	**%**
Children who had undergone surgery	60	96.8%
Number of surgeries per participant (min-max)	4.0 (1-21)	
Ear reconstruction	24	38.7%
Skin tag removal	24	38.7%
Tympanostomy tubes and tympanoplasty	20	32.3%
Adenoidectomy and/or tonsillectomy	17	27.4%
Dental restoration/extraction	16	25.8%
Bone anchored hearing aid abutment surgery	13	21.0%
Aural atresia repair	8	12.9%
Gastrostomy tube placement/removal	6	9.7%
Lower jaw surgery	6	9.7%
Lateral oral cleft surgery	6	9.7%
Other ophthalmologic surgery	6	9.7%
Removal of epibulbar dermoid	5	8.1%
Cleft lip and/or cleft palate surgery	4	6.5%
Cardiac surgery	3	4.8%
Tracheostomy surgery	3	4.8%
Coloboma surgery	2	3.2%
Fat graft surgery	2	3.2%
Nerve surgery	2	3.2%
Speech surgery	2	3.2%
Urologic surgery	2	3.2%
LeFort I advancement	1	1.6%
Rhinoplasty or septoplasty	1	1.6%
Other surgery*	18	29.0%

*Other surgery includes other uncommon procedures such as abscess incision and drainage

### Composition of “Chapters”

Most caregivers organized their story according to key moments in their child's CFM treatment journey, such as “Diagnosis”, “Meeting the Specialists”, “First BAHA”, and “Surgery”. Others named their chapters according to broad time periods, for example, “Birth”, “The First Year”, “Kindergarten”, and “School”. Several caregivers focused their chapters on the emotions they experienced at each time point, such as “Confused and Scared”, “Sad and Frustrated”, “Happiness and Anguish” and “Hope and Positivity”. Some caregivers used creative phrases to name their chapters, for example, “The Short End of the Stick”, “A New Beginning”, “The Little Things Matter” and “Hitting our Stride”. Caregivers of 3-11-year-olds identified an average of 5 chapters (range = 2-9) and focused in detail on the early years. Caregivers of 12-17-year-olds identified an average of 6 chapters (range = 3-14), which spanned the whole of childhood.

### Components of the Framework

**Figure 1.**
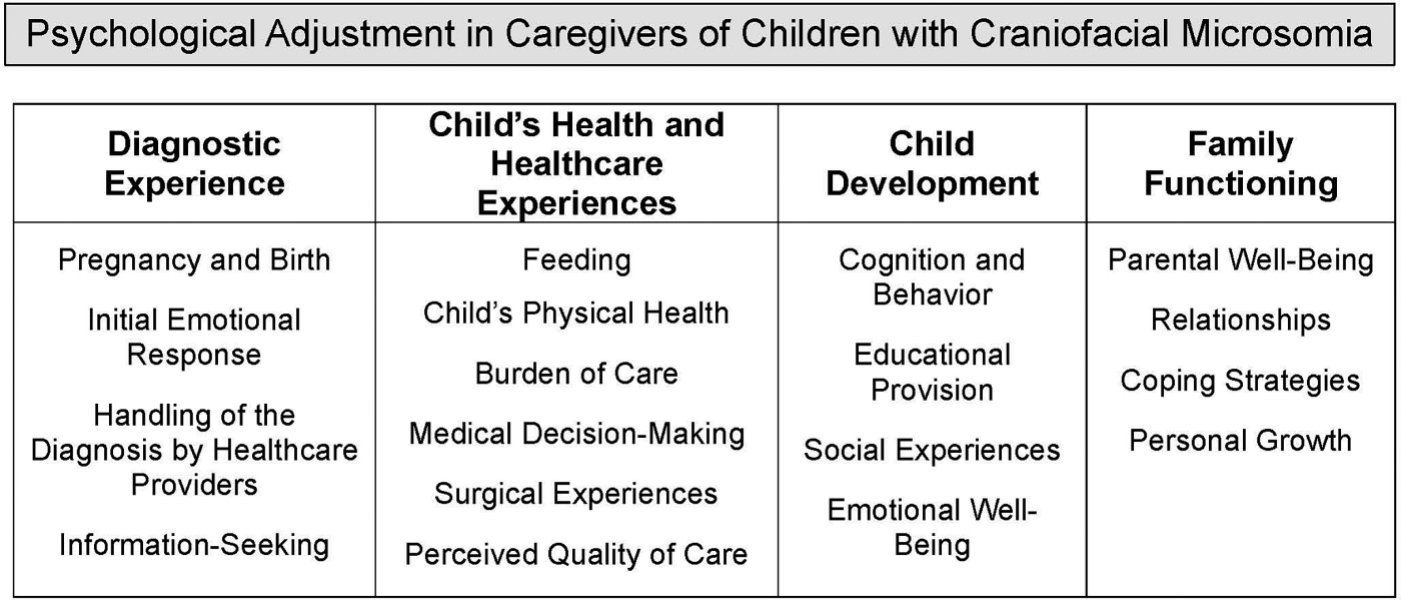
A conceptual thematic framework of psychological adjustment in caregivers of children with craniofacial microsomia.

Themes and subthemes were collated into a conceptual thematic framework to illustrate the components that caregivers identified as meaningful in adjusting to their child's CFM (Figure 1). Each of these components is briefly summarized below.

#### Diagnostic Experience

##### Pregnancy and Birth

Few participants had experienced significant complications during pregnancy, and prenatal concerns were only identified occasionally. Most participants’ narratives therefore began at the birth of their child, which could be described as either broadly positive (despite there being something unknown and different about their child), or characterized by a considerable degree of distress, panic, and confusion. Participants particularly missed the opportunity to hold their baby before they were taken away for screening or medical care.

##### Initial Emotional Response

Participants reported a range of emotions in response to seeing their baby for the first time. This included joy, love, shock, anxiety, anger, shame, guilt, and worry for the future, alongside feeling overwhelmed and symptoms of depression. Often, several of these emotions were experienced simultaneously.

##### Handling of the Diagnosis by Healthcare Providers

While some participants described a neutral or fairly positive diagnostic experience, many reported a scarcity of information that evoked significant anxiety. Negative interactions with healthcare providers were reported, including insensitive language and unprofessional curiosity about the child's condition. In contrast, healthcare providers who were well-informed, warm, offered reassurance to participants that they were not responsible for their child's diagnosis, and instilled hope about the future were highly appreciated by participants. Initial evaluations and newborn screening tests were described as incessant and overwhelming by most, with a minority perceiving this degree of assessment to be reassuring.

##### Information-Seeking

Given that participants commonly reported receiving little to no information from healthcare providers after the birth, most sought information about their child's condition independently. The majority of English-speaking participants looked for information online, with some participants also seeking information from family or friends with medical backgrounds. While many participants felt empowered in this process, others struggled to navigate the disjointed and occasionally frightening information they came across.

#### Child's Health and Healthcare Experiences

##### Feeding

Participants reported a number of challenges associated with CFM features that prevented them from feeding their child as they had hoped. Some mothers felt shamed for not being able to breastfeed their child and were given few alternative feeding options until it became evident that the child was failing to thrive. Most participants had to try multiple feeding methods and sources of nutrition to adapt to their child's complex needs. Difficulties related to digestion, gastroesophageal reflux, and feeding tubes were also reported. Feeding challenges were a cause of considerable distress and frustration for both caregivers and infants, particularly as participants often struggled to obtain feeding-related information and/or support from healthcare providers.

##### Child's Physical Health

The child's physical health was a source of ongoing stress for participants. Specific physical health concerns focused heavily on hearing, as well as issues related to speech, vision, breathing, preauricular/facial tags, sleep, growth, renal, cardiac, dentistry/orthodontics, and facial paralysis, many of which required additional screening, medical monitoring, and/or medical interventions or surgeries. Participants also reported considerable relief during periods of relatively stable health.

##### Burden of Care

Due in part to the lack of knowledge surrounding CFM, participants struggled to obtain referrals to appropriate specialists. Participants frequently spoke about the need to be strong advocates in order for their child to access appropriate care. In many cases, identifying a craniofacial team had been a lengthy and complex process, often involving long wait times. At the time of interview, not all families were affiliated with a craniofacial team. Furthermore, participants described the frequency of medical appointments, and the travel and time off work necessary to attend visits, particularly when local services were lacking. Some families had chosen to relocate, sometimes across states, to be closer to a hospital and/or to access what they perceived to be the best care. Health insurance and the financial implications of healthcare also featured heavily in participants’ narratives.

##### Medical Decision-Making

Participants embarked on a decision-making process years before specific treatments became an option for their child. Treatment motivations were frequently related to real and/or anticipated social reactions to their child's visible difference, and/or concerns related to hearing, education, and other future prospects (such as gaining meaningful employment). Participants sought authoritative guidance from healthcare providers, as well as the opinions of family members, peers, and English-language online communities. Practical issues, such as insurance coverage, were also considered. Participants offered different views regarding their child's capacity and role in the decision-making process. Following treatment, participants reflected on whether they had made the “right” decision. These reflections were influenced by the degree to which participants’ expectations of treatment were met and any complications that had occurred, which in turn impacted their views of future treatment.

##### Surgical Experiences

Participants described a significant amount of anxiety in the lead up to a surgery, even if participants considered the surgery to be minor. Seeing their child undergo anesthesia was a particularly difficult experience for participants, coupled with the need to place their faith in the hands of the healthcare providers. Participants also worried about the impact of surgeries on their child's physical health (including recovery) and psychological well-being. Participants’ anxiety was lessened by reassurance from staff prior to and during surgery, well-coordinated pre- and post-operative care, and perceived successful surgical outcomes. Completion of surgeries was often viewed as surpassing a milestone and making significant progress on the treatment journey.

##### Perceived Quality of Care

Throughout their child's healthcare journey, participants continued to report difficult interactions with healthcare providers who lacked knowledge of CFM and the treatment pathway. Further, a lack of care coordination and inconsistencies in the provision of medical information over time were repeatedly mentioned. In contrast, healthcare providers who were clear in their communication, were empathic, were sensitive to cultural needs, possessed specialist knowledge, and shared additional resources throughout the family's journey were highly valued. Overall, participants were satisfied with the care they had received from craniofacial specialists.

#### Child Development

##### Cognition and Behavior

Some participants reported their child to have multiple developmental needs, with some children having received a specific developmental diagnosis (such as Attention-Deficit/Hyperactivity Disorder). Additional challenges included balance or coordination difficulties, which could affect day-to-day life and specific activities, such as playing sports. Many children had difficulties locating sounds, which was particularly problematic in the classroom. Hearing difficulties were partially rectified by hearing aids/implants, although some participants reported that these devices were ineffective, uncomfortable, or upsetting for their child, which posed challenges for adherence. Children with visual impairments also struggled to wear glasses if they could not effectively rest their glasses on their ears. Participants perceived that these difficulties negatively affected their child's cognitive functioning and behavior, as well as their own well-being, yet also reported feeling encouraged when their child reached developmental milestones.

##### Educational Provision

Overall, participants believed that attending school had a positive impact on their child's socioemotional development and learning, which in turn improved caregiver well-being. Participants were proud of their child's educational achievements. Nonetheless, many participants’ children had educational needs, and participants had to make concerted efforts to ensure that appropriate support was provided for their child. These provisions (including preferential seating, hearing assistive technology, and Individualized Education Programs) had a positive impact when used well, yet participants felt frustrated and exhausted when the school failed to implement these measures consistently. A minority of participants had chosen to home-school their child, either during long periods of treatment recovery, or due to social difficulties, such as bullying.

##### Social Experiences

Participants frequently worried about the impact of their child's visible difference on their child's social experiences. These worries typically centered on their child being asked intrusive questions, being teased or bullied, or being socially excluded. Participants proactively used different approaches to address their concerns, such as holding a school assembly and/or sending letters to other parents at the school to raise awareness of CFM. Some also encouraged their child to join after-school clubs or play sports, taught their child gestures and American Sign Language to increase communication opportunities, and spoke to their child about how to respond to comments and questions about their condition. Concerns about the child's social inclusion were lessened if participants perceived their child to have a good friendship group and/or that the school promoted an inclusive environment. Participants also gained confidence if their child began to successfully navigate social experiences.

##### Emotional Well-Being

Some participants expressed concerns about their child's psychological well-being, including anxiety, symptoms of depression, and the psychological impact of treatment. In a minority of cases, participants reported symptoms of eating disorders and/or suicidality in their child. These concerns were a source of considerable stress for caregivers. Consequently, participants invested strongly in developing their child's social confidence, self-esteem, and adaptive coping skills. This included treating their child the same as their other children, choosing not to hide their child's CFM features, encouraging open communication about CFM, following their child's lead, and suppressing the desire to be overprotective.

#### Family Functioning

##### Parental Well-Being

Having a child with CFM had a significant and ongoing impact on the psychological and physical well-being of participants and other family members and required them to continually adjust to the demands placed on them. Participants reported varying presentations of anxiety, symptoms of depression, guilt, frustration, shame, and feeling overwhelmed, in addition to stress reactions to their child's medical interventions. The burden of their child's care and the weight of the responsibility associated with this was often described as physically and emotionally exhausting. Additional life stressors beyond their child's condition impacted participants’ ability to cope, such as moving home, changing jobs, divorce, or a death in family. Participants reflected on the choices they had made for their child throughout their journey and hoped that they had supported their child in the best way possible.

##### Relationships

The demands of their child's condition also significantly impacted participants’ relationships with others. Some participants described difficulties and/or a breakdown in their relationship with their partner, in addition to conflicts within the family and social isolation from friendship groups. In contrast, support from partners, extended family, and friends was highly valued, as was the opportunity to connect with other families affected by CFM, either online or in person. Some participants had chosen to proactively share their experiences with other families and/or advocacy groups to support others affected by CFM. As their child grew older, some participants reported that it became more difficult to talk to their child about the impacts of CFM and its treatment, which put a strain on the caregiver-child relationship. Others discussed how they felt a strengthened bond with their child as they moved through CFM journey together.

##### Coping Strategies

Caregivers spoke extensively about the different coping strategies they used to help them adjust to their child's condition and its associated challenges. These included positive reframing, making comparisons to families who were less fortunate, talking with others about their experiences, journaling, drawing on their faith, calling on friends and family for help, and focusing on practical steps they could take to support their child.

##### Personal Growth

Reflecting on their journey as a whole, participants were gratified for having negotiated the demands of having a child with CFM, often reporting personal growth as a result. Participants described having gained more confidence and inner strength, alongside self-efficacy as a parent, and a belief that they could competently address future challenges.

### Highs and Lows

Participants identified a series of “high” and “low” points from their experiences of caring for their child with CFM (Table 4). “Low” points encompassed a period of adjusting to the diagnosis, concerns about the child's physical health and development, managing healthcare demands, arranging effective educational provisions, and concerns for the family's psychological health. “High” points encompassed caregivers’ love for their child, positive experiences of healthcare and community support, seeing their child thrive, and personal and familial growth.

**Table 4.** “Low” Points and “High” Points of the CFM Journey Identified by Caregivers.

Theme	“Low” Points	“High” Points
Diagnostic Experience	Complications during pregnancy Difficult birth Reduced opportunities to hold the baby after birth Overwhelming and conflicting emotions Delay in getting a diagnosis Not understanding the etiology of CFM Self-blame Lack of information/communication Inaccurate/inconsistent information Lack of support/insensitivity from hospital staff Uncertainty about the future	Joy at birth of new baby Positive interactions with healthcare providers Receiving a clear and timely diagnosis Bonding with child
Child’s Health and Healthcare Experiences	Child being admitted to the NICU Being unable to breastfeed Concerns about child's physical health/hospital readmission(s) Child experiencing hearing difficulties/complications with hearing devices Searching for care/accessing care Financial implications of healthcare Burden of care Needing to be a strong advocate to achieve progress Experiencing pressure to “fix” their child Child ineligible for certain treatments Complex emotions involved in medical decision-making Surgical complications/need for further surgery Caregiver stress reactions to child's medical treatment Decisional regret	Confirmation of no additional medical problems Establishing an effective feeding plan Acquiring an effective hearing device Identifying a specialist craniofacial team Access to local services Periods of health stability and fewer appointments/interventions Feeding tube removal Getting past surgical “hurdles” Satisfaction with the outcome of medical decisions and treatment
Child Development	Difficulties accessing early intervention Challenging experiences with teachers Concerns about the child's behavior Separation anxiety Child becoming aware of their “difference” Caregiver deliberately concealing child's “difference” Child experiencing hurtful comments from peers/social exclusion School transitions Child exhibiting signs of depression, anxiety, social withdrawal, medical traumatic stress and suicidality	Child achieving developmental milestones/exceeding expectations Positive school culture and accommodations Child enjoying school Child making friends Child engaging in activities Child beginning to advocate for themselves Child beginning to accept their “difference” Accessing good psychological support
Family Functioning	Caregiver postpartum depression Strain on marital relationship/divorce Child's siblings experiencing disruption Family separation during periods of inpatient care Negative impact on caregivers’ employment and careers Difficult social interactions Strain on caregiver-child relationship Feeling helpless as a parent Ongoing concerns about the future	Becoming connected to a peer support network Becoming closer as a family Strengthened marital bond Increased self-efficacy in managing child's needs Personal growth as a caregiver

### Research Priorities

Participants identified 11 priorities for future research, which were ranked in order of frequency (Table 5). The highest priorities include the need to offer integrated psychological support (rank #1) and provide reliable information (rank #2) from birth onward. Caregivers also prioritized research to clarify the etiology of CFM and what to expect from the treatment pathway over time (rank #3).

**Table 5.** Research Priorities Identified by Caregivers and Ranked by Frequency.

Ranking	Research Priorities to Improve Care for CFM
1	Psychological support for families and children from birth onwards
2	Information to help families navigate the treatment journey
3	Understanding CFM etiology and prognosis
4	Developing an evidence-based treatment pathway for CFM
5	Improving health provider knowledge and public awareness of CFM
6	Support with medical decision-making
7	Advances in technology and surgical techniques
8	Improving the physical health of children with CFM
9	Improving access to specialist care
10	Support for children with CFM in schools
11	Improving prenatal detection of CFM

### Participants’ Reflections on the Interview Process

Toward the end of the interview, participants were asked to reflect on the narrative interview process. Some participants found the unstructured nature of the narrative approach a little daunting and had struggled to decide how to tell their story and/or which parts of their journey to focus on. This was also reflected in observations that some participants came prepared, while others chose their chapters during the interview itself. Some participants stated they found it emotionally challenging to relive some of their more difficult experiences. Nonetheless, participants expressed that they were glad their experiences were being valued, and some described the process as ‘therapeutic’. They enjoyed the unique opportunity to tell their story from start to finish, reflecting on how far they had come on their journey, and found the narrative approach to be a useful way of organizing their ideas.

## Discussion

Having a child with healthcare needs can have a significant and long-term impact on the psychosocial well-being of caregivers and families.^4,5,7,8,24,25^ This paper brings together the components of the caregiver CFM journey, as identified and recounted in detail by 62 US caregivers, and as illustrated in a new conceptual thematic framework to guide future CFM research and practice.

Across the narratives collected, caregivers described an overarching and continuous process of adjusting to the demands of their child's condition. These reported experiences reflect existing reviews of parental adjustment to craniofacial conditions,^26,27^ as well as broader models of parental stress and coping in the context of chronic illness and/or disability.^28-31^ According to these models, and rather than representing a singular event, chronic medical conditions typically initiate a series of events in which families experience various highs and lows, interact with a myriad of healthcare providers, and must learn to manage their child's fluctuating healthcare needs.^31^ In addition, caregivers must interact with multiple systems, including the healthcare and education systems, as well as local services, to ensure their child's needs are met.^32^ Drawing on these various models, the caregiver experience can therefore be shaped by the varying demands placed on the family, the appraisals made by the family about the experienced and potential impacts of CFM, the internal and external resources available, and the coordinated and individual coping strategies employed.^28-31^ The findings of the current study are discussed below, situated within the context of existing broad models of caregiver adjustment, to inform future work in this area.

## Demands

In line with previous research,^4^ having a child with healthcare needs represented a significant demand, which most caregivers were not expecting. From the point of birth, caregivers had to adjust their expectations of parenthood and cope with a considerable degree of uncertainty due to the rare nature of their child's condition.^33^ The number of required screening tests, evaluations by subspecialty healthcare providers, and interventions were significant stressors for caregivers, alongside the ongoing need to navigate treatment decisions and assume the overall burden of care.^4^ Additionally, caregivers were observant of their child's fluctuating ability to cope with CFM-related demands.

## Appraisals

In light of the demands placed on them, caregivers made appraisals about what having a child with CFM meant for them and their family.^28^ Caregivers assessed the potential impact of CFM according to their perceptions of sociocultural expectations (eg, their child being seen as ‘different’); their child's ability to engage with education, physical activities, and social situations; and the ability of medical and surgical interventions to reduce these burdens and increase their child's chances of success. Caregiver appraisals were influenced by sociodemographic factors,^30^ the responses of significant others and members of the public to their child's diagnosis, interactions with healthcare providers, the quality of the information and support received, and families’ treatment experiences.

## Resources

Caregivers drew on a range of external resources to help them address the demands associated with CFM. Predominantly, this included support from healthcare providers. While healthcare providers who were perceived as empathic and knowledgeable positively influenced caregiver well-being, difficult or uninformative interactions with providers were often distressing, and caregivers described long-lasting negative impressions. This was also true for difficult interactions with local service providers and education providers. Caregivers’ ability to access appropriate services and treatments/devices, obtain sufficient health insurance coverage, and implement school supports effectively all impacted caregiver well-being and family functioning. Given the reliance that caregivers have on these providers to support their child's development,^32^ the success or failure of these external systems can have a considerable impact on the family's ability to cope with demands. Previous research in craniofacial^10,15,34^ and other chronic health conditions^4,33^ has consistently emphasized the importance of healthcare satisfaction and appropriate educational provisions for caregiver well-being and family functioning, and has called for improvements in healthcare provider knowledge, as well as reliable information provision, access to early intervention, and integrated psychological support for families.

A second key external resource was social support. This support often came in the form of friends, family, partners, local community, and/or online/in-person peer support networks (such as advocacy groups^13,16^). Social support could be emotional or practical in nature, and included encouragement, advice, childcare support, and help to attend medical appointments. In line with prior craniofacial research,^10^ caregiver well-being was negatively impacted if social support was perceived to be lacking, and/or if there was conflict within the marital relationship.

## Coping

Caregivers drew on a range of internal resources and coping strategies to help them address CFM-related demands. Many caregivers took on an advocacy role, which included independent information-seeking, keeping their own medical records, navigating insurance issues, pushing for access to various services, and educating others about CFM. While the responsibility of managing their child's needs was significant, some caregivers also found it empowering to adopt a proactive approach.^35^ Caregivers often adapted their parenting strategies to their child's specific needs, with the aim of helping their child to navigate CFM-related challenges successfully. Other internal resources utilized by caregivers, such as positive reframing, drawing on their faith, and emotional expression, align with previous CFM and broader research^12,30^ and offer insight into the types of intervention that could facilitate caregiver well-being. Coping was negatively impacted by co-occurring life stressors, such as job insecurity, separation/divorce, and/or a death in the family, indicating that additional support to manage CFM-related demands may be necessary during these times.

## Adjustment

Psychological adjustment to illness involves not only the prevention of emotional distress, but the fostering of resilience and personal growth.^30,36^ Positive adjustment depends on having insight into the changes that have occurred as the result of a difficult event(s), acceptance of these changes, the acquisition of helpful coping strategies, appropriate modification of beliefs and personal goals, and the re-establishment of important relationships.^37^ Caregivers considered themselves to be well-adjusted if they had successfully navigated the demands placed on them to date, felt they had grown personally as a result, believed they had the ability to tackle future demands, and were optimistic about the future.

## Future Research

While this paper provides a broad overview of the CFM journey, the field would also benefit from learning about caregivers’ experiences in each of these areas in more detail. The CARE team therefore intends to further explore caregivers’ qualitative reports, in addition to the narratives of young people and adults affected by CFM. Longitudinal qualitative research may also offer insight into how caregiver experiences change over time and add to the findings from the current study. Future quantitative investigation of the proposed framework would allow for exploration of relationships between the components, and the identification of risk and protective factors for psychological distress. Future research can also be guided by the priorities identified by caregivers themselves, presented in this and other related papers.^15^ Of particular value is examination of interventions to promote psychological well-being in families, alongside high-quality information from birth onward, and a better understanding of CFM etiology and long-term prognosis.

The components of the conceptual thematic framework for caregiver adjustment to CFM correspond well to an existing framework developed in the related area of cleft lip and/or palate.^38^ Findings are also similar to those presented in comprehensive reviews of caregiver adjustment to their child's diagnosis of a rare disorder,^33^ developmental disability,^5^ and chronic illness.^4^ Similar findings across conditions emphasize an overlap in the psychosocial and treatment experiences of caregivers of children with chronic medical conditions, and implies the use of similar measures and interventions may be beneficial.^39^ Further exploration of existing psychological interventions to support caregiver well-being and the applicability to the CFM community is warranted.

## Methodological Considerations

Limitations of this study include the largely well-educated, employed, White/European American sample, which may not represent the experiences of populations with fewer resources. Fathers’ experiences were also underrepresented, reflecting an ongoing need for greater participation by fathers in pediatric research.^40^ Efforts were made to capture the experiences of Spanish-speaking caregivers, whose broad experiences aligned with English-speaking participants. As well as being integrated into the conceptual thematic framework, the unique aspects of the Spanish-speaking participants’ experiences are also reported in a separate paper (*under review*). The second phase of the CARE program will examine the applicability of individual components of the framework across additional countries and cultures. The narrative approach used in this study relied on caregiver recall and report. Nonetheless, caregivers found the narrative approach to be a useful and enjoyable way of sharing their story, often for the first time. This narrative approach allowed caregivers the freedom to direct their own story and to identify the aspects of their story they considered important.

## Conclusions

This paper offers a summary of the core components of the CFM caregiver journey, drawn directly from caregivers’ own narratives. This work has enabled the development of a conceptual thematic framework to guide clinical care and future research. Children's medical history and caregiver treatment narratives emphasize the burden of care and highlight several areas for the improvement of care delivery. Psychological support for families and provision of reliable information about CFM and all treatment options are critical areas of focus for future research.

## Supplemental Material

sj-jpg-1-cpc-10.1177_10556656241245284 - Supplemental material for A Conceptual Thematic Framework of Psychological Adjustment in Caregivers of Children with Craniofacial Microsomia Supplemental material, sj-jpg-1-cpc-10.1177_10556656241245284 for A Conceptual Thematic Framework of Psychological Adjustment in Caregivers of Children with Craniofacial Microsomia by Nicola M. Stock, DPhil, Bruna Costa, DHealthPsy, Jade Parnell, PhD, Alexis L. Johns, PhD, ABPP, Canice E. Crerand, PhD, Kristin Billaud Feragen, Clin. Psychol., PhD, Laura P. Stueckle, MPH, Angela Mills, Leanne Magee, PhD, Matthew Hotton, DClinPsy, Melissa Tumblin, Amy Schefer, Amelia F. Drake, MD, and Carrie L. Heike, MD, MS in The Cleft Palate Craniofacial Journal

## References

[1] Birgfeld C Heike C . Craniofacial microsomia. Clin Plast Surg. 2019;46(2):207-221. 30851752 10.1016/j.cps.2018.12.001

[2] Renkema RW ERN CRANIO Working Group on Craniofacial Microsomia. European guideline craniofacial microsomia. J Craniofac Surg. 2020;31(Suppl 8):2385-2484 32804824 10.1097/SCS.0000000000006691

[3] American Cleft-Palate Craniofacial Association. Parameters for evaluation and treatment of patients with cleft lip/palate or other craniofacial differences. Updated 2018. https://acpa-cpf.org Web site. https://journals.sagepub.com/doi/pdf/10.1177/1055665617739564. 10.1177/1055665617739564 34162066

[4] Cousino MK Hazen RA . Parenting stress among caregivers of children with chronic illness: a systematic review. J Pediatr Psychol. 2013;38(8):809-828. doi:10.1093/jpepsy/jst049 23843630

[5] Masefield SC Prady SL Sheldon TA Small N Jarvis S Pickett KE . The caregiver health effects of caring for young children with developmental disabilities: a meta-analysis. Matern Child Health J. 2020;24(5):561-574. doi:10.1007/s10995-020-02896-5 32048172 PMC7170980

[6] Melnyk BM Feinstein NF Moldenhouer Z Small L . Coping in parents of children who are chronically ill: strategies for assessment and intervention. Pediatr Nurs. 2001;27(6):548-558. 12024526

[7] Cohn LN Pechlivanoglou P Lee Y , et al. Health outcomes of parents of children with chronic illness: a systematic review and meta-analysis. J Pediatr. 2020;218:166-177.e2. doi:10.1016/j.jpeds.2019.10.068 31916997

[8] Bayer ND Wang H Yu JA Kuo DZ Halterman JS Li Y . A national mental health profile of parents of children with medical complexity. Pediatrics. 2021;148(2):e2020023358. doi:10.1542/peds.2020-023358 34155129

[9] Sischo L Clouston SAP Phillips C Broder HL . Caregiver responses to early cleft palate care: a mixed method approach. Health Psychol. 2016;35(5):474-482. doi:10.1037/hea0000262 26280177 PMC4757521

[10] Stock NM Costa B White P Rumsey N . Risk and protective factors for psychological distress in families following a diagnosis of cleft lip and/or palate. Cleft Palate Craniofac J. 2020;57(1):88-98. doi:10.1177/1055665619862457 31378083

[11] Costa B Edwards W Wilkinson-Bell K Stock NM . Raising a child with craniosynostosis: psychosocial adjustment in caregivers. Cleft Palate Craniofac J. 2023;60(10):1284-1297. doi:10.1177/10556656221102043 35786018

[12] Johns AL Stock NM Costa B Billaud Feragen K Crerand CE . Psychosocial and health-related experiences of individuals with microtia and craniofacial microsomia and their families: narrative review over 2 decades. Cleft Palate Craniofac J. 2023;60(9):1090-1112. doi:10.1177/10556656221091699 35382590 PMC10803131

[13] Johns AL Luquetti DV Brajcich MR Heike CL Stock NM . In their own words: caregiver and patient perspectives on stressors, resources, and recommendations in craniofacial microsomia care. J Craniofac Surg. 2018;29(8):2198-2205. doi:10.1097/SCS.0000000000004867 30334912 PMC6224304

[14] Johns AL Im DD Lewin SL . Early familial experiences with microtia: psychosocial implications for pediatric providers. Clin Pediatr (Phila). 2018;57(7):775-782. 28959893 10.1177/0009922817734358

[15] Luquetti DV Brajcich MR Stock NM Heike CL Johns AL . Health care and psychosocial experiences of individuals with craniofacial microsomia: patient and caregivers perspectives. Int J Pediatr Otorhinolaryngol. 2018;107:164-175. doi:10.1016/j.ijporl.2018.02.007 29501301 PMC5839339

[16] Umbaugh HM Crerand CE Stock NM , et al. Microtia and craniofacial microsomia: content analysis of Facebook groups. Int J Pediatr Otorhinolaryngol. 2020;138:110301. doi:10.1016/j.ijporl.2020.110301 32838996

[17] Kancherla V Romitti PA Damiano PC Druschel CM Robbins JM . Maternal reports of satisfaction with care and outcomes for children with microtia. Plast Reconstr Surg. 2009;123:149e-150e. 10.1097/PRS.0b013e31819e5bea PMC6042857 19337070

[18] Nelson PA . Qualitative approaches in craniofacial research. Cleft Palate Craniofac J. 2009;46(3):245-251. doi:10.1597/08-121.1 19642761

[19] Stock NM Crerand CE Johns AL , et al. Establishing an international interdisciplinary research network in craniofacial microsomia: the CARE program. Cleft Palate Craniofac J. 2023:10556656231176904. doi:10.1177/10556656231176904 PMC10984877 37248561

[20] Riessman C . Narrative methods for the human sciences. Sage; 2008.

[21] McAdams DP . The psychology of life stories. Rev Gen Psychol. 2001;5(2):100-122.

[22] Birgfeld CB Luquetti DV Gougoutas AJ , et al. A phenotypic assessment tool for craniofacial microsomia. Plast Reconstr Surg. 2011;127(1):313-320. doi:10.1097/PRS.0b013e3181f95d15 21200224

[23] Braun V Clarke V . Thematic analysis: a practical guide. Sage; 2022.

[24] McPherson M Arango P Fox H , et al. A new definition of children with special health care needs. Pediatrics. 1998;102(1 Pt 1):137-139. doi:10.1542/peds.102.1.137 9714637

[25] Coleman CL Morrison M Perkins SK Brosco JP Schor EL . Quality of life and well-being for children and youth with special health care needs and their families: a vision for the future. Pediatrics. 2022;149(Suppl 7):e2021056150G. doi: 10.1542/peds.2021-056150G 35642872

[26] Nelson PA Caress AL Glenny AM Kirk SA . Doing the “right” thing’: how parents experience and manage decision-making for children’s ‘normalising’ surgeries. Social Science Med. 2012;74(5):796-804. 10.1016/j.socscimed.2011.11.024 22305806

[27] Feragen KB Stock NM . Psychological adjustment to craniofacial conditions (excluding oral clefts): a review of the literature. Psychol Health. 2017;32(3):253-288. doi:10.1080/08870446.2016.1247838 27925479

[28] Folkman S Lazarus RS . The relationship between coping and emotion: implications for theory and research. Soc Sci Med. 1988;26(3):309-317. doi:10.1016/0277-9536(88)90395-4 3279520

[29] McCubbin M McCubbin H . Resiliency in families: a conceptual model of family adjustment and adaptation in response to stress and crises. In: McCubbin HI Thompson AI McCubbin MA , eds. Family assessment: resiliency, coping and adaptation - inventories for research and practice. University of Wisconsin; 1996:1-64.

[30] Walsh F . Family resilience: a developmental systems framework. European Journal of Developmental Psychology. 2016;13:313-3324. doi:10.1080/17405629.2016.1154035

[31] Didericksen KW Muse A Aamar R . Rethinking parental coping with child health: a proposed theoretical model. Marriage Fam Rev. 2019;55(5):423-446. doi:10.1080/01494929.2018.1501631

[32] Bronfenbrenner U . Toward an experimental ecology of human development. American Psychologist. 1977;32(7):513-531. doi:10.1037/0003-066X.32.7.513

[33] von der Lippe C Neteland I Feragen KB . Children with a rare congenital genetic disorder: a systematic review of parent experiences. Orphanet J Rare Dis. 2022;17(1):375. doi:10.1186/s13023-022-02525-0 36253830 PMC9575260

[34] Stock NM Ridley M . Young person and parent perspectives on the impact of cleft lip and/or palate within an educational setting. Cleft Palate Craniofac J. 2018;55(4):607-614. 29554456 10.1177/1055665617734991

[35] Feragen KB Stock NM Myhre A Due-Tønnessen BJ . Medical stress reactions and personal growth in parents of children with a rare craniofacial condition. Cleft Palate Craniofac J. 2020;57(2):228-237. 31426676 10.1177/1055665619869146

[36] Revenson T Hoyt M . Chronic illness and mental health. In: Howard S Friedman (Ed). Encyclopedia of Mental Health. 2016:284-292. Elsevier: Oxford. doi:10.1016/B978-0-12-397045-9.00151-8

[37] Beaumont JG . Chapter 27 - clinical neuropsychology in rehabilitation. In: Stokes M , ed. Physical management in neurological rehabilitation (second edition). Elsevier Mosby; 2004:461-468.

[38] Stock NM Zucchelli F Hudson N Kiff JD Hammond V . Promoting psychosocial adjustment in individuals born with cleft lip and/or palate and their families: current clinical practice in the United Kingdom. Cleft Palate Craniofac J. 2020;57(2):186-197. doi:10.1177/1055665619868331 31431061

[39] Stock NM Feragen KB . Comparing psychological adjustment across cleft and other craniofacial conditions: implications for outcome measurement and intervention. Cleft Palate Craniofac J. 2019;56(6):766-772. doi:10.1177/1055665618770183 29652532

[40] Ferreira de Moura A Philippe K . Where is the father? Challenges and solutions to the inclusion of fathers in child feeding and nutrition research. BMC Public Health. 2023;23. doi:10.1186/s12889-023-15804-7 PMC10280827 37337169

