# Supplementary figures and images for: A Conceptual Thematic Framework of Psychological Adjustment in Caregivers of Children with Craniofacial Microsomia
Source: Cleft Palate Craniofac J. 2024 Apr 8;62(7):1228–40. doi: 10.1177/10556656241245284 (PMC11458819; doi:10.1177/10556656241245284)

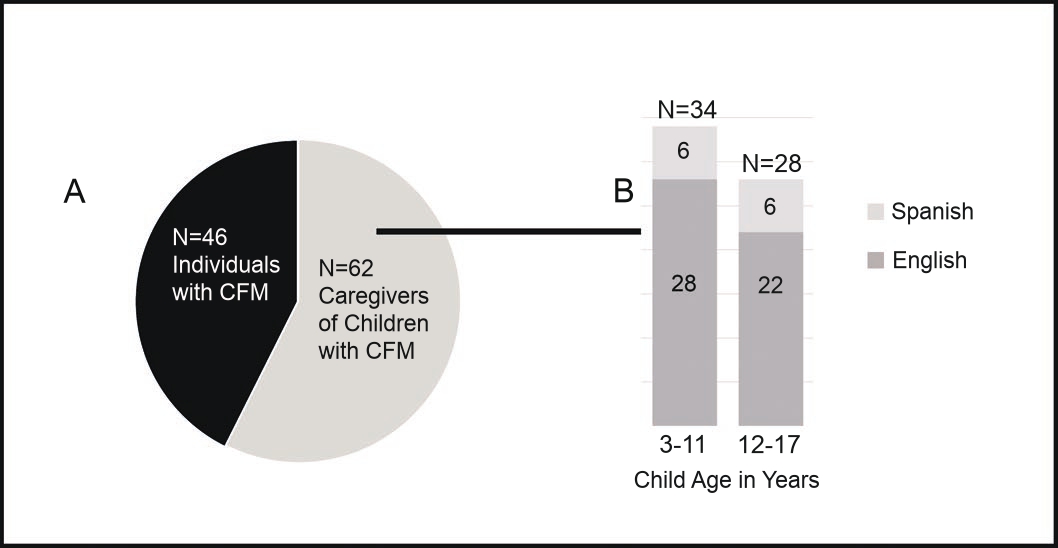

Supplement: sj-jpg-1-cpc-10.1177_10556656241245284 - Supplemental material for A Conceptual Thematic Framework of Psychological Adjustment in Caregivers of Children with Craniofacial Microsomia [file sj-jpg-1-cpc-10.1177_10556656241245284.jpg]
